# Estimating the burden of influenza‐associated hospitalization and deaths in Oman (2012‐2015)

**DOI:** 10.1111/irv.12500

**Published:** 2017-12-05

**Authors:** Doaa M. Abdel‐Hady, Rima M. Al Balushi, Badr A. Al Abri, Seif S. Al Abri, Hanan S. Al Kindi, Amina K. Al‐Jardani, Fatma M. Al Yaqubi, Idris S. Al Abaidani

**Affiliations:** ^1^ Department of Communicable Diseases Ministry of health Muscat Oman; ^2^ Faculty of Medicine Mansoura University Mansoura Egypt; ^3^ Directorate General for Diseases Surveillance & Control Ministry of Health Muscat Oman; ^4^ Central Public Health Laboratory Ministry of Health Muscat Oman

**Keywords:** burden of disease, influenza in‐hospital death, influenza‐associated hospitalization, Oman, severe acute respiratory infections

## Abstract

**Background:**

Influenza is a serious vaccine‐preventable disease with high incidence, hospitalization, and mortality in high‐risk groups. The epidemiology, seasonality, and risk factors for influenza are well defined in most of the temperate countries, but estimating influenza burden in the World Health Organization (WHO) Region for the Eastern Mediterranean is scarce. In Oman, despite the advancements in influenza surveillance, the clinical burden and seasonality of influenza remain not fully understood.

**Objectives:**

To estimate the incidence of influenza‐associated hospitalizations and in‐hospital death in Oman.

**Patients and methods:**

Influenza‐associated hospitalizations and in‐hospital deaths were estimated using hospital discharge records based on ICD‐10 codes (J09‐J18), results of virological analysis and population census for the period between 2012 and 2015.

**Results:**

During 2012 and 2015, we identified a total of 19 405 influenza‐associated hospitalization and 847 deaths. Influenza positivity percentage ranged from 6.4% in 2013 to 20.6% in 2015. Influenza‐associated hospitalization incidence rate was 7.3 (95% CI: 6.4‐8.1) per 100 000 in 2013 and 27.5 (95% CI: 25.9‐29.1) per 100 000 in 2015 with an overall rate of 20.6 (95% CI: 19.9‐21.3) per 100 000. The highest incidence of influenza‐associated death was among those aged ≥65 years and ranged between 39.5 (95% CI: 27.3‐51.8) per 100 000 in 2014 and 11.3 (95% CI: 7.5‐15.1) in 2015.

**Conclusions:**

Influenza causes a substantial number of hospitalizations and deaths in Oman. Hospitalization rates were highest among children, and adults ≥65 years showed the highest death rate. The potential value of using seasonal influenza vaccine in these groups should be considered.

## 1 INTRODUCTION

Influenza is believed to be an important cause of morbidity and mortality worldwide. It is a vaccine‐preventable disease that annually affects 5%‐10% of the population globally.^1^ It causes approximately 1 million influenza‐associated hospitalizations among children younger than 5 years and 500 000 deaths among all age groups.^2^, ^3^

The wide spectrum of clinical presentation and outcomes of influenza^4^ and the low care seeking level with ill‐defined symptoms that are often treated empirically without respiratory sampling make it difficult to estimate the true burden of influenza.^5^ However, it is very important to document the clinical burden of influenza in order to reveal the pattern of severe illness, and to guide public health policies and justify the costly investment in the seasonal influenza vaccination,^6^ and to assess influenza vaccine impact^7^ which remains the key preventive intervention for influenza‐related hospitalizations and deaths.

Influenza‐associated hospitalization has been studied in many countries around the world,^8^, ^9^ but such estimate remains scarce, especially in the World Health Organization (WHO) Region for the Eastern Mediterranean.

The investment in epidemiology and laboratory capacity during recent years to face many emerging and re‐emerging diseases from respiratory origin, for example, severe acute respiratory syndrome (SARS), Middle East respiratory syndrome corona virus (MERS‐CoV), and novel influenza pathogens such as H5N1 and influenza pandemics, substantially improved surveillance and data quality about acute respiratory infections.^10^

Oman is a country situated in Southwest Asia, between Yemen, United Arab Emirates (UAE), and the Kingdom of Saudi Arabia. The country has an area of 309 500 square kilometers and is composed of varying topographic features: valleys and desert account for 82 percent of the land mass; mountain ranges, 15 percent; and the coastal plain, 3 percent. The weather is a hot dry interior, humid coastal strip, and a mountainous southern region with seasonal (May‐September) monsoon rainfall.^11^ Oman is divided into eleven governorates/ provinces with total 60 wilayats. The Sultanate of Oman has a total population of 4 588 683 with about 45.9% expatriates.^12^ Oman is considered a high‐income country according to the World Bank list of economies.^13^

In Oman, a computerized online system named “Nabdh Al Shifa” is established and has direct access to patient records and generating data from the 11 regional hospitals. The reports are generated through software (COGNOS) in response to input of International Classifications of Disease (ICD‐10 code). The ICD‐10 codes/J course is related to respiratory diseases and ICD‐10 codes (J09‐J18) are corresponding to patients hospitalized with pneumonia or influenza which are the WHO proxies for severe acute respiratory infections (SARI).^14^

The objective of this study was to estimate the incidence of influenza‐associated hospitalizations and in‐hospital death in Oman.

## 2 PATIENTS AND METHODS

Hospital discharge and in‐hospital death based on ICD‐10 codes (J09‐J18), results of virological analysis, and population census data were retrieved for the period between January 1, 2012 and December 31, 2015 to estimate influenza‐associated hospitalizations and in‐hospital deaths using a mathematical formula used in previous study with the same purpose.^5^

### 2.1 Calculating the number of SARI and in‐hospital death

Although, SARI surveillance was developed in Oman since 2008 in four sentinel sites using WHO case definition of SARI. This study was based on ICD‐10 codes (J09‐J18) which are the WHO proxies^14^ for SARI to estimate the national burden of influenza.

Hospital discharge records based on ICD‐10 codes (ICD‐10 codes J09‐J18) and in‐hospital death data generated from directorate general of information technology in the ministry of health were examined. For each month during the study period (2012‐2015), the number of patients hospitalized and in‐hospital death corresponding to ICD‐10 codes (J09‐J18) were calculated and were stratified by age group, into <1 year, 1‐<5 years, 5‐<15 years, 15‐<50, 50‐<65 years, and ≥65.

### 2.2 Calculating influenza positivity

Results of virological testing using real‐time reverse transcription polymerase chain reaction (RT‐PCR) for the respiratory tested samples collected from SARI patients were retrieved from the National Influenza Center (NIC) influenza surveillance line list. The age‐stratified influenza positivity proportion was calculated for each month of the studied period by dividing the number of influenza positive samples by the total tested sample in the same month.

We excluded from the analysis 2.5%‐5% of cases due to duplication, rejected samples, results pending, missing age group or samples requested for non‐influenza.

### 2.3 Calculating the population at risk based upon population census

The population at risk was considered as the total population as it is assumed that each individual was susceptible for developing SARI that requires admission in a public hospital. The number of population was provided from the National Center for Statistics and Information (NCSI) and stratified by year and age group.

### 2.4 Estimating the burden

The incidence of influenza‐associated hospitalization and death was estimated as the sum of products of the number of the age‐stratified monthly hospital discharge and in‐hospital death based on ICD‐10 codes (J09‐J18) by the monthly influenza positivity of respiratory samples withdrawn from the corresponding age group. This estimated monthly age‐stratified number of influenza‐associated hospitalizations and deaths was divided by the number of midyear population in that age group (population at risk). We calculated 95% confidence intervals (95% CI) assuming a Poisson distribution.

### 2.5 Ethical issues

All data sources used in this study were anonymous records, and personal identifiers were deleted. And as analyzed data are part of the ARI surveillance, according to ethical regulations, it was exempt of IRB review.

## 3 RESULTS

The age structure of the population remained almost the same throughout the study period, that is, those aged <1 year represented 1.7% while the ≥65 years were <4% of the total population (Table 1)

**Table 1:** Estimated National influenza‐associated hospitalizations and deaths in Oman 2012‐2015

Year	Age group (y)	Population size^a^	Severe acute respiratory infection		Annual percentage of influenza positivity^d^	Estimated influenza‐associated (per 100 000)	
			Hospitalizations^b^	Mortality^c^		Hospitalizations^e^ rate (95% CI)	Deaths^f^ rate (95% CI)
2012	<1	63 183 (1.7%)	1059 (26.3%)	14 (10.3%)	7.9%	148.6 (118.6‐178.7)	2.5 (−1.4‐6.4)
	1‐<5	259 658 (7.2%)	1218 (30.3%)	3 (2.2%)	14.9%	59.1 (49.8‐68.5)	0.2 (−0.3‐0.7)
	5‐<15	476 292 (13.1%)	449 (11.1%)	4 (2.9%)	17.2%	16.3 (12.7‐20.0)	0.2 (−0.2‐0.5)
	15‐<50	2 464 564 (68.0%)	486 (12.1%)	14 (10.3%)	28.2%	4.5 (3.6‐5.3)	0.1 (0.0‐0.3)
	50‐<65	263 314 (7.3%)	263 (6.5%)	10 (7.3%)	32.6%	27 (20.7‐33.3)	0.8 (−0.3‐1.9)
	≥65	95 990 (2.6%)	549 (13.6%)	91 (6.6%)	29.5%	111.1 (90.0‐132.2)	19.1 (10.3‐27.8)
	Total	3 623 001 (100%)	4024 (100%)	136 (100%)	269/1427 (18.8%)	19.0 (17.6‐20.5)	0.7 (0.4‐1)
2013	<1	66 504 (1.7%)	1233 (29.9%)	9 (4.5%)	2.8%	61.0 (42.2‐79.8)	1.1 (−1.3‐3.6)
	1‐<5	276 627 (7.2%)	1177 (28.5%)	4 (2.0%)	3.7%	19.7 (14.5‐24.9)	0.1 (−0.3‐0.6)
	5‐<15	492 100 (12.8%)	421 (10.2%)	4 (2.0%)	5.7%	5.1 (3.1‐7.1)	0.1 (−0.2‐0.3)
	15‐<50	2 645 447 (68.6%)	450 (10.9%)	24 (12.0%)	12.3%	1.9 (1.4‐2.5)	0.1 (0.0‐0.3)
	50‐<65	275 916 (7.1%)	280 (6.8%)	34 (17.0%)	11.3%	12.1 (8.0‐16.2)	1.4 (0.0‐2.7)
	≥65	98 604 (2.6%)	568 (13.8%)	125 (62.5%)	9.7%	53.4 (38.9‐67.8)	12.6 (5.6‐19.6)
	Total	3 855 198 (100%)	4129 (100%)	200 (100%)	175/2703 (6.4%)	7.3 (6.4‐8.1)	0.4 (0.2‐0.5)
2014	<1	71 009 (1.8%)	1338 (25.8%)	11 (4.8%)	5.4%	113.1 (88.4‐137.9)	1.1 (−1.3‐3.6)
	1‐<5	290 786 (7.3%)	1528 (29.4%)	6 (2.7%)	11.6%	61.5 (52.5‐70.5)	0.3 (−0.3‐0.9)
	5‐<15	522 888 (13.1%)	465 (9.0%)	5 (2.2%)	16.1%	12.4 (9.4‐15.4)	0.1 (−0.2‐0.4)
	15‐<50	2 718 234 (68.1%)	686 (13.2%)	16 (7.0%)	25.6%	5.9 (5.0‐6.8)	0.1 (0.0‐0.3)
	50‐<65	288 751 (7.2%)	450 (8.7%)	31 (13.8%)	23.2%	34.5 (27.7‐41.3)	2.7 (0.8‐4.5)
	≥65	101 225 (2.5%)	723 (14.0%)	161 (70.9%)	21.6%	182.8 (156.5‐209.1)	39.5 (27.3‐51.8)
	Total	3 992 893 (100%)	5190 (100%)	230 (100%)	903/5654 (15.9%)	19.7 (18.3‐21.2)	0.9 (0.6‐1.2)
2015	<1	75 057 (1.7%)	1744 (28.8%)	14 (5.0%)	7.2%	175.1 (145.2‐20.50)	1.7 (−1.2‐4.7)
	1‐<5	306 600 (7.0%)	1911 (31.5%)	7 (2.5%)	17.6%	106.3 (94.7‐117.8)	0.3 (−0.3‐1.0)
	5‐<15	546 957 (12.3%)	502 (8.3%)	2 (0.7%)	20.0%	25.0 (20.8‐29.1)	0.1 (−0.2‐0.5)
	15‐<50	2 819 813 (64.9%)	610 (10.1%)	42 (15.0%)	29.9%	6.2 (5.3‐7.2)	0.4 (0.2‐0.7)
	50‐<65	302 338 (6.9%)	399 (6.6%)	35 (12.4%)	32.5%	40.8 (33.6‐48.0)	3.5 (1.4‐5.7)
	≥65	295 819 (6.8%)	896 (14.7%)	181 (64.4%)	22.3%	62.2 (53.2‐71.1)	11.3 (7.5‐15.1)
	Total	4 346 584 (100%)	6062 (100%)	281 (100%)	1515/7357 (20.6%)	27.5 (25.9‐29.0)	1.2 (0.9‐1.5)
2012‐2015	<1	275 801 (1.7%)	5374 (27.7%)	48 (5.7%)	5.9%	121.3 (108.4‐134.3)	1.1 (−0.1‐2.4)
	1‐<5	1 133 671 (7.2%)	5834 (30.0%)	20 (2.7%)	13.2%	71.7 (66.8‐76.6)	0.3 (0.0‐0.6)
	5‐<15	2 038 237 (12.8%)	1837 (9.5%)	15 (1.7%)	29.9%	6.2 (5.3‐7.2)	0.4 (0.2‐0.7)
	15‐<50	10 648 058 (67.4%)	2232 (11.5%)	96 (11.3%)	25.7%	5.3 (4.9‐5.8)	0.2 (0.1‐0.3)
	50‐<65	1 130 319 (7.1%)	1392 (7.2%)	110 (13.0%)	26%	32.1 (28.8‐35.4)	2.2 (1.4‐3.1)
	≥65	591 638 (3.7%)	2736 (14.1%)	558 (65.8%)	20.4%	92.3 (84.6‐100.1)	18.6 (15.1‐22.1)
	Total	15 817 724 (100%)	19 405 (100%)	847 (100%)	2862/17 141 (16.7%)	20.6 (19.9‐21.3)	0.9 (0.7‐1.0)

a population census based on National Center for Statistics and Information by age group for Omanis and non‐Omanis.

b Number of persons hospitalized during the year with severe acute respiratory infection (SARI) proxy diagnoses (ICD‐10 codes J9‐18).

c Number of deaths among persons hospitalized during the year with SARI proxy diagnoses (ICD‐10 codes J9‐18).

d Annual number respiratory samples positive for influenza through PCR over total number tested (percentage). (Not used in the formula).

e Sum of the products of the proportion of samples testing positive for influenza and the number of persons hospitalized with by age group and month. Rate per 100 000 population.

f Sum of the product of the proportion of samples testing positive for influenza and the number of persons dying by age group and month. Rate per 100 000 population.

During the study period (2012‐2015) in Oman, a total of 19 405 SARI cases that correspond to (ICD‐10 J09‐J18) were identified and 847 related deaths. Children aged 1‐<5 years comprised 30% of the total hospitalizations while the greatest proportion of in‐hospital deaths occurred among those aged ≥65 years (65.8%). (Table 1).

**Figure 1:**
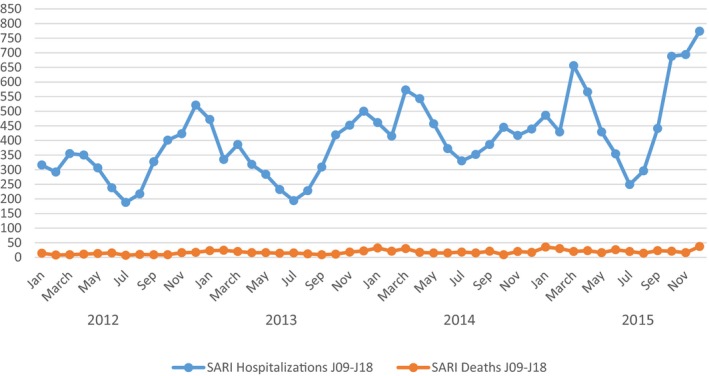
Monthly number of severe acute respiratory infections (SARI) hospitalization and deaths and influenza positivity rate (2012‐2015)

The number of SARI hospitalization and SARI‐related deaths (ICD‐10 J09‐J18) throughout the entire study period were plotted in Figure 1. The peak of SARI activity (hospitalization) varied across years. It peaked in November‐December 2012 and 2013, in February‐March 2014, and in 2015, it had 2 peaks in February‐March and November‐December.

Nationwide, a total of 17 141 respiratory samples were tested (2862 were positive for influenza ‐ average influenza positivity percentage: 17%). Influenza positivity for the whole study period ranged between 5.9% in diagnosed infants <1 year and 29.9% in patients aged 5<15 years. Also, influenza annual positivity rate showed difference between years with the highest rates of total positivity in 2015 (20.6%) and the lowest in 2013 (6.4%) (Table 1).

**Figure 2:**
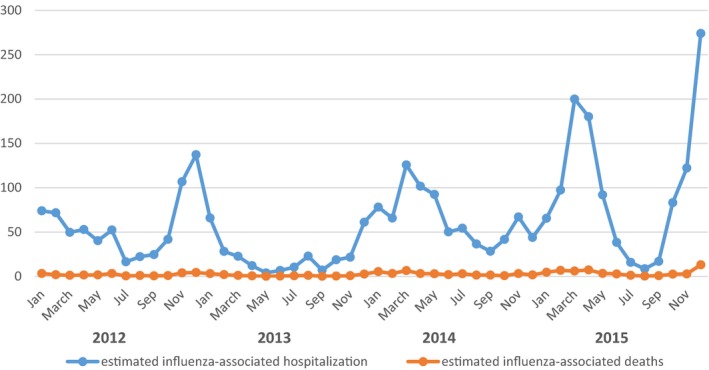
Estimated monthly number of influenza‐associated hospitalization and deaths (2012‐2015)

The incidence of influenza‐associated hospitalization in the study period was 20.6 (95% CI: 19.9‐21.3) per 100 000 population; it ranged between 7.3 (95% CI: 6.4‐8.1) per 100 000 population in 2013 and 27.5 (95% CI: 25.9‐29.1) per 100 000 population in 2015. The highest annual incidence of influenza‐associated hospitalization was found among infants <1 year of age in all years of the study except in 2014, where the highest incidence rate occurred among those aged ≥65 years. Cumulative incidence of influenza‐associated hospitalization calculated for all the study period (2012‐2015) among those <1 year was 121.3 (95% CI: 108.4‐134.3) per 100 000; this was about 24 folds greater than the lowest incidence calculated among those 15<50 years: 5.3 (95% CI: 4.9‐5.8) per 100 000 (Table 1); Figure 2.

People aged ≥65 years had the highest annual incidence of influenza‐associated in‐hospital death in all the study years and ranged between 11.3 (95% CI: 7.5‐15.1) in 2015 and 39.5 (95% CI: 27.3‐51.8) per 100 000 population in 2014. The lowest cumulative influenza‐associated in‐hospital death was observed for those aged 15‐<50 years; 0.2 (95% CI: 0.1‐0.3) per 100 000. (Table 1)

**Figure 3:**
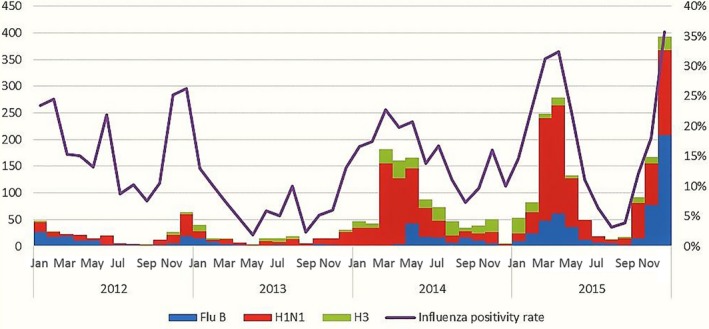
Number of specimen positive for influenza, influenza subtyping, and influenza positivity rate (2012‐2015)

In addition, the monthly influenza positivity percentage was as low as 2% in January and October 2013 and reached 36% in November 2015 (Figure 3). Different peaks for influenza activity were detected in different years. It peaked (influenza activity = 25%) in December in 2012, and peaked in February in 2013 and 2014 (13% and 23% consequently), and it peaked in November in 2015. Influenza A (H1N1pdm09) was the predominant subtype in all the years (53% in 2012, 63% in 2013, 64.2% in 2014, and 59% in 2015 of total influenza cases). (Figure 3).

## 4 DISCUSSION

This is the first study in Oman to estimate the clinical burden (incidence rate of morbidity and mortality per 100 000 population) of influenza on the national level.

During the study period, which lasts for 4 years, neither influenza immunization strategy nor age distribution changed.

The results show that influenza caused a substantial morbidity and mortality in Oman, and during the study period, it is estimated that it lead to 3253 influenza‐associated hospitalizations and 142 deaths.

However, it is very difficult to compare our results with other studies for many reasons. For example, rates in different studies were generated using different case definitions, age grouping, surveillance, durations, seasons, and analytical methods. Moreover, similar studies in the neighboring Eastern Mediterranean countries are very few. Also, different influenza immunization policies in different countries may affect the outcome of these estimations.

The estimated annual incidence rate of influenza‐associated hospitalization across all age groups between 2012 and 2015 (ranged 7.3‐27.5 per 100 000 person) and this is much higher than a previous study in Oman^15^ based on a sentinel sites surveillance between 2008 and 2013 that estimated the annual incidence rate across all age groups from 0.5 to 15.4 cases per 100 000 person.

On the other hand, the overall estimated rate of influenza‐associated hospitalization in this study (20.6 per 100 000 person 95% CI: 19.9‐21.3) is much lower than the burden of influenza in one of the Egyptian Districts^16^ in 2013 where the overall incidence of influenza virus‐associated SARI during the study period was estimated to be 44 cases per 100 000 person‐years (95% CI: 39‐48).

Our results are more consistent with those reported from Iran where the overall incidence of influenza‐associated SARI in three provinces was estimated at 29.0 per 100 000 population.^17^

Findings regarding the age distribution of estimated incidence of morbidity and mortality in this study were consistent with other studies reported all over the world. For example, our results showed that very young children (<1 year) were most commonly hospitalized as a result of influenza. A meta‐analysis study^2^ estimated the global burden of influenza in young children reported that highest incidence of influenza episodes, influenza‐associated acute lower respiratory infections, and influenza‐associated severe aLRI occurred in the first year of life. Another study estimated the incidence of influenza‐associated hospitalizations using the same tool as in our study in 5 countries of Central America showed the highest distribution of influenza‐associated hospitalization in children aged <5 years.^5^

Death rates calculated in this study were lower than those estimated for deaths from pneumonia associated with influenza in Brazil (1.4, 95% CI 0.7‐2.1/100 000 person),^18^ and deaths attributed to influenza‐associated respiratory illness in Mexico (3.7 [95% CI 3.0‐4.4]/100 000 population).^19^ These differences might be explained by the different patterns of healthcare seeking behavior or access to healthcare services.

Influenza‐associated mortality in our study was most frequently among adults aged ≥65 years. And this was similar to many other studies.^5^, ^18^, ^20^, ^21^, ^22^, ^23^ The estimated influenza‐associated in‐hospital death rates among adults ≥65 years ranged between 7.3 per 100 000 population as estimated in Central America^5^ and 29.0 per 100 000 population as reported in a study conducted in Iran^17^ compared to 18.6 per 100 000 population in our study.

Although different years showed difference in influenza peaking, transmission of influenza viruses was seen throughout the year, with a decrease in influenza activity observed during September of all years. This pattern of seasonality is comparable to that of other countries in our Region^16^ and in a previous study conducted in Oman.^15^

H1N1pdm09 was the predominant influenza virus in all seasons and this was in contrast with some studies that reported A (H3N2) virus as 54% in 2013,^16^ but similar to a study in Jordan where H1N1pdm09 was the predominant in 2012‐2013.^24^

This study has some limitations. First, SARI‐ICD data in this study were obtained from the Health information and statistics department generating data from all Ministry of Health (MoH) institutions, but SARI data for non‐MoH institutions are not included in our study estimate (represents about 1%).

The second limitation is that the age grouping in this study is not matched with that recommended by WHO and used in many studies as it was based on the groups available by Health information and statistics department. Thus, one of the significant age groups (0‐<2 years) was distributed between 2 groups <1 year and 1‐<5 years. This limits the capacity of the study to describe the epidemiological picture of SARI and seasonal influenza in this important age group and hinders the comparison of our results with other studies.

Also, the specific selection of patients only under ICD‐10 codes (J09‐J18) as SARI “than selecting the whole J codes (respiratory diseases)” may lead to underestimation of the burden because not all influenza cases are recorded under ICD‐10 codes (J09‐J18) and sometimes recorded as codes related to other respiratory diseases or exacerbation of comorbidities. Finally, the burden of influenza‐associated mortality may be underestimated as it reflects only the in‐hospital deaths associated with influenza adding to that the common poor documentation of death causes in developing countries.

## 5 CONCLUSIONS AND RECOMMENDATIONS

Despite limitations, this study is the first one providing a preliminary estimation for the clinical burden of influenza in Oman on the national level throughout the period of 4 years. Our findings suggest that the potential value of introducing seasonal influenza vaccines for children and the elderly is substantial. Further stratification of the first age group (<1 year) to 0‐<6 and ≥6 months is recommended to give a practical view for vaccination strategy in this age group. Additional work is needed for better estimates of the influenza burden in risk groups other than age‐based groups, for example, pregnant and associated comorbidity in order to support informed decision‐making regarding the selection of high priority groups for targeting seasonal influenza vaccination.
